# circRNA: Regulatory factors and potential therapeutic targets in inflammatory dermatoses

**DOI:** 10.1111/jcmm.17473

**Published:** 2022-06-29

**Authors:** Ruifeng Liu, Luyao Zhang, Xincheng Zhao, Jia Liu, Wenjuan Chang, Ling Zhou, Kaiming Zhang

**Affiliations:** ^1^ Shanxi Key Laboratory of Stem Cells for Immunological Dermatosis, Institute of Dermatology Taiyuan Central Hospital of Shanxi Medical University Taiyuan China; ^2^ Huashan Hospital, Shanghai Medical College Fudan University Shanghai China

**Keywords:** circRNA, dermatoses, inflammation, skin

## Abstract

The skin is the largest organ of the human body and acts as the first line of defence against injury and infection. Skin diseases are among the most common health problems and are associated with a considerable burden that encompasses financial, physical and mental consequences for patients. Exploring the pathogenesis of skin diseases can provide insights into new treatment strategies. Inflammatory dermatoses account for a large proportion of dermatoses and have a great impact on the patients' body and quality of life. Therefore, it is important to study their pathogenesis and explore effective treatment. Circular RNAs (circRNAs) are a special type of RNA molecules that play important regulatory roles in several diseases and are involved in skin pathophysiological processes. This review summarizes the biogenesis, properties and functions of circRNAs as well as their roles in the pathogenesis of inflammatory dermatoses, including psoriasis, lupus erythematosus, atopic dermatitis, lichen planus and severe acne and their potential as therapeutic targets.

## 1 INTRODUCTION

The skin, which forms an interface between the body and the environment, provides the first line of defence against exogenous insults. Skin disorders not only cause considerable economic burden to patients, but also negatively impact their physical and mental health. For example, both severe acne and psoriasis can negatively impact the quality of life. Therefore, understanding the pathogenesis of skin diseases is crucial to the development of therapeutic approaches. Circular RNAs (circRNAs) are a special type of RNA molecule involved in several diseases and can serve as biomarkers or therapeutic targets. For instance, circRNAs are diagnostic markers and potential therapeutic targets for lung, breast and colorectal cancers.^1^ circRNAs also play a role in the pathological process of various cardiovascular diseases, such as myocardial infarction, heart failure and atherosclerosis.^2^ Associations of circRNAs with autoimmune diseases and viral infections have also been reported.^3^ In addition, circRNAs are involved in the pathogenesis of skin diseases through various molecular mechanisms. This review discusses the biological origin, properties and functions of circRNAs as well their roles in inflammatory skin disorders.

## 2 circRNA

A circRNA is a non‐coding RNA derived from back‐splicing, a type of non‐canonical splicing in eukaryotes. Different from linear RNAs, circRNAs form a closed ring structure with covalent bonds, which is more stable against exonucleases. Increasing evidence has proposed a regulatory role for circRNAs in gene expression.

### 2.1 Brief research history of circRNAs

In 1971, ‘viroids’ were found in potato and they were the first circRNA molecules to be discovered.^4^ And, the ultrastructure of circRNAs was first observed using an electron microscope in 1976. Several circRNAs have since been discovered in abnormal splicing transcripts of some genes, including the tumour suppressor gene *DCC* in humans, testis‐determining gene *Sry* in mice and the cytochrome P450 *2C24* gene in rats. Supported by high‐throughput RNA sequencing, numerous circRNAs in eukaryotes, including plants, fungi, protists and various types of metazoans, have been identified, and their abundance exceeds that of their associated linear mRNA in some cases, suggesting that circRNAs are old, universal and conserved in the gene expression programmes of eukaryotes. CircRNAs were believed to result from splicing errors with unknown function at first. In 1988, Weiner et al.^5^ found that circRNAs with open reading frames encode the antigenic polypeptides P24 Delta and P27 Delta in hepatitis delta virus. Later studies showed that circRNAs can act as microRNA (miRNA) sponges via their binding sites, regulating the activity of miRNAs on certain target genes. More recently, the involvement of circRNAs in the physiology and pathology of various tissues has also been revealed.

### 2.2 Biogenesis of circRNAs

**FIGURE 1:**
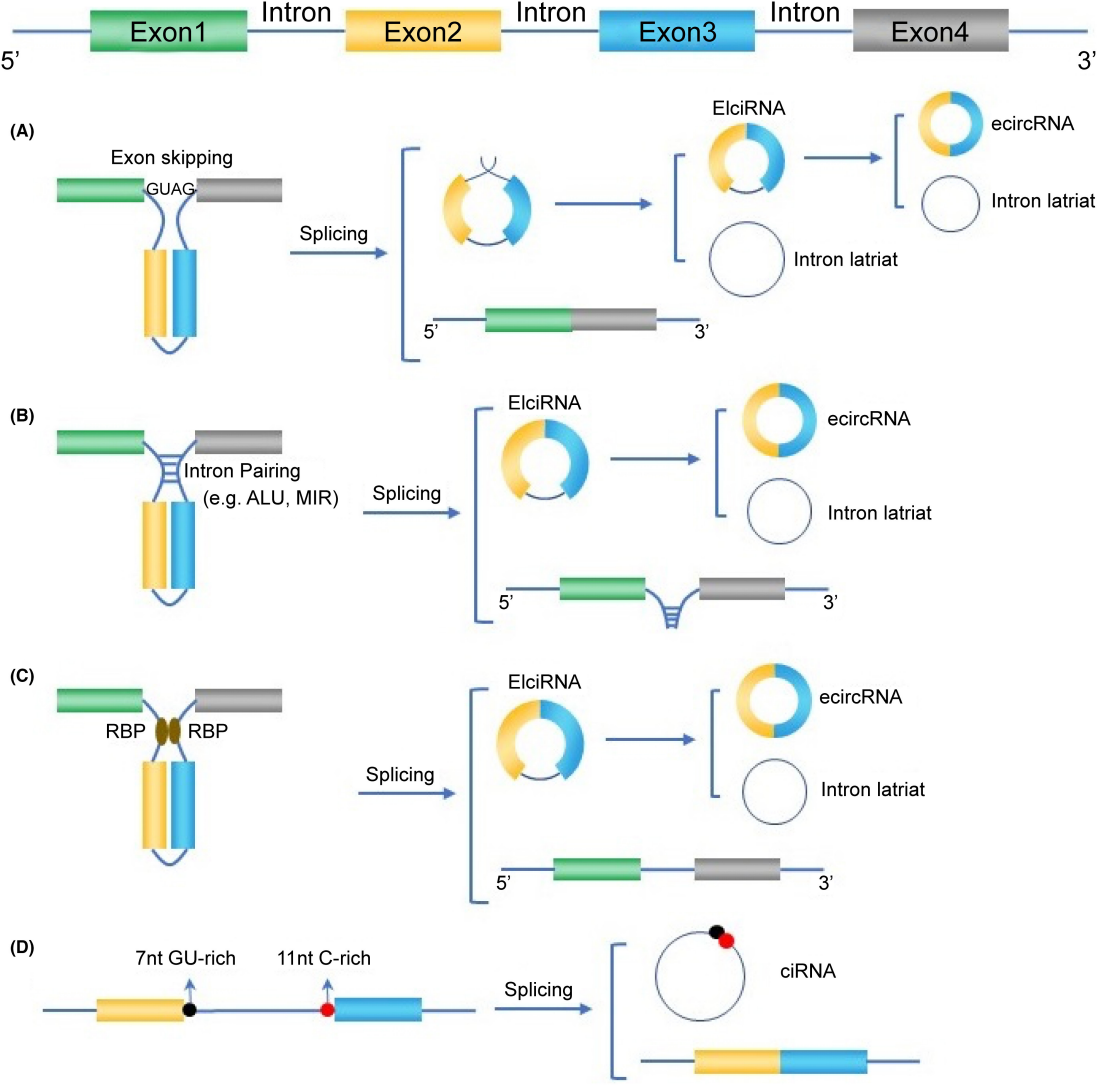
Biogenesis of circRNA. (A) Lariat‐driven circularization. The pre‐mRNA is folded and spliced, resulting in exon‐skipping, followed by removal or retention of the introns to form ecircRNA or EIciRNA. (B) Intron‐pairing‐driven circularization. Base pairing of long flanking complementary introns next to the exons is circularized to form ecircRNA or EIciRNA. (C) RBP‐driven circularization. RBPs binding to sequence motifs of flanking introns interact with each other to form a bridge to facilitate circularization, leading to the formation of ecircRNA or EIciRNA. D. Biogenesis of ciRNAs. The 7nt GU‐rich element at the 5′‐end (black circle) and the 11nt C‐rich element at the 3′‐end (red circle) help intron lariats escape debranching and become stable ciRNAs

The circRNAs are generally categorized into exonic circRNAs (ecircRNAs),^6^ exon‐intron circRNAs (EIciRNAs),^7^ intronic circRNAs (ciRNAs)^8^ and intergenic circRNAs.^9^ EIciRNAs and ciRNAs are predominantly present in the nucleus, whereas ecircRNAs are mainly enriched in the cytoplasm. As previously mentioned, circRNAs are derived from back‐splicing, a type of alternative splicing that occurs via different mechanisms from those of canonical linear splicing. The 3′ splicing donor downstream can covalently bind to the 5′ splicing receptor upstream in reverse order. The possible mechanisms underlying the biogenesis of circRNAs are discussed below and illustrated in Figure 1.

#### 2.2.1 Lariat‐driven circularization

Evidence indicates that the production of circRNAs is a result of exon‐skipping events. The pre‐mRNA is spliced in accordance with the classical GU‐AG rule. Owing to the folded pre‐mRNA, the non‐adjacent exons are at close proximity to each other, resulting in exon‐skipping. After back‐splicing, a lariat precursor containing exons and introns is formed, which can splice itself internally to remove introns and form an ecircRNA. However, EIciRNA is produced when introns are not completely spliced but remain in the newly generated circRNAs (Figure 1A).^10^

#### 2.2.2 Intron‐pairing‐driven circularization

In the mechanism of intron‐pairing‐driven circularization, back‐splicing to a circular molecule is likely triggered by intronic sequences adjacent to the exons that will be circularized. The intronic sequences can interact with each other, bringing the ends of exons into close proximity. Long flanking complementary introns are essential for the formation of circRNAs, and these sequences are usually either non‐repetitive complementary sequences or intronic repeat sequences, including Alu elements and mammalian‐wide interspersed repeats.^11^, ^12^ Back‐splicing is driven by base pairing of these sequences. Similar to lariat‐driven circularization, a lariat precursor containing both exons and introns, or EIciRNA, is formed after back‐splicing, followed by the removal of introns to form an ecircRNA. In this model, certain structures such as Alu elements bring non‐adjacent sequence pairs into apposition, initiating circularization, without the requirement of exon‐skipping (Figure 1B).^13^

#### 2.2.3 RNA‐binding protein (RBP)‐driven circularization

Another mechanism for circRNA formation based on RBPs has been proposed. The splicing factors, including heterogeneous nuclear ribonucleoprotein L (HNRNPL),^14^ Quaking (QKI),^15^ Muscleblind (MBL),^13^ FUS^16^ and immune factor NF90/NF110,^17^ can bind to certain motifs in the flanking introns on both sides of the exon, which will be back‐spliced, bringing these introns into close proximity with each other and facilitating the generation of circRNAs. The main difference between this process and intron‐pairing‐driven circularization is that the former is regulated by RBPs while the latter relies on complementary sequence pairing (Figure 1C).

#### 2.2.4 Other mechanisms

Biogenesis of ciRNAs relies on a motif that consists of a 7 nucleotide (nt) element with enriched GU ribonucleotides at the 5′ splicing end and an 11 nt element with enriched C ribonucleotides at the 3′ end near the branchpoint site. Generally, intron lariats are degraded by debranching enzymes, but the above structures can prevent such enzymes from binding to the ciRNAs (Figure 1D).^8^

#### 2.2.5 Regulation of circRNA biogenesis

During circRNA biogenesis, several regulators can influence the biogenesis rate. It has been demonstrated that the transcription elongation rate (TER) of a gene is influenced by whether it can produce circRNA; if a gene can produce circRNA, its TER will be high.^18^ A mild increase in TER promotes circRNA formation, as fast elongation pairs non‐adjacent complementary sequences across one or more exons and introns,^18^ consequently promoting circularization. The spliceosome is also associated with the rate of circRNA formation. Depletion or inhibition of some core spliceosome components, such as SF3b and SF3a complexes, increases the expression of circRNAs while decreasing the expression of their associated linear mRNAs,^19^ suggesting a possible conversion from canonical alternative splicing to back‐splicing. Moreover, some sequences, such as simple poly(A) sequences and mild distortions like G‐U wobble pairs, can suppress circRNA circularization.^11^ Although several mechanisms of circRNA biogenesis have been identified, the precise mechanisms remain unclear.

### 2.3 Properties of circRNAs

#### 2.3.1 Stable molecules

The circRNAs of eukaryotic cells are exceptionally stable.^20^ Because circRNAs have no free 3′ poly(A) tail or 5′ cap, they are resistant to RNase R, a potent 3′‐to‐5′ exonuclease that can efficiently degrade linear RNAs.^21^ The half‐life of mRNAs is 10 h on average, whereas most exonic circRNA species have a half‐life of over 48 h, which can be attributed to their resistance to RNA exonucleases.^22^

#### 2.3.2 Diversity and specific expression patterns

More than 25,000 circRNAs have been identified in human fibroblasts.^23^ At the individual gene level, one gene locus can produce multiple circRNAs via various mechanisms. Generally, circRNA expression is low, but some circRNAs are expressed at higher levels than those of their linear mRNAs.^24^ For example, circRNA expression increases substantially compared with that of linear isoforms during central nervous system ageing. Hence, circRNAs can serve as ageing biomarkers in *Drosophila*. Under certain circumstances, the amount of circRNAs can be over 10‐fold richer than that of the corresponding linear mRNAs. Additionally, circRNA expression is tissue‐ and cell‐type‐specific. For example, AMBRA1 RNA shows differential expression of two types of circular isoforms in different cells; the long isoform (362 nt) is expressed at higher levels in MCF‐7 cells, whereas the short isoform (182 nt) shows higher expression in HepG2 cells. Moreover, the mammalian brain is enriched with circRNAs,^25^ and in *Drosophila*, circMbl expression in the brain is considerably higher than in the ovary.^26^ The circRNA expression also varies with the developmental stage. During human epithelial–mesenchymal transition, the expression of circRNAs markedly increases.^15^ This phenomenon has also been reported in the mouse brain, where the expression of 258 circRNAs in the cortex and 250 circRNAs in the hippocampus was higher in 22‐month‐old mice than in 1‐year‐old mice.^27^

#### 2.3.3 Evolutionary conservation and variation between different species

Studies have found that some circRNAs in humans are also present in the circRNA expression profile of mice, with 10%–20% being identical.^28^ In fact, 457 human circRNAs can be mapped to murine genes, 69 of which contain homologous back‐splice points. The complementary sequences in introns near the back‐splice points, instead of the circRNA sequences themselves, are associated with the emergence of these conserved circRNAs. Moreover, compared with species‐specific circRNAs, the conserved molecules are more abundant in both humans and mice.^28^

### 2.4 Functions of circRNAs

Although circRNA expression in eukaryotes is generally very low, many play regulatory roles in gene expression under various physiological and pathological conditions, such as during the innate immune response, basic functioning of nerve cells and cell differentiation and proliferation.

#### 2.4.1 Acting as miRNA sponges

Competing endogenous RNAs (ceRNAs) can bind miRNAs via specific sites, acting as miRNA sponges and regulating miRNA function on their target genes.^29^ As a type of ceRNA, circRNAs, such as *CDR1as* or *ciRS‐7*,^30^ circ‐*HIPK3*,^31^ circ‐*SRY*, circRNA_010383,^32^ hsa_circ_0000615^33^ and hsa_circ_0000735,^34^ can also act as sponges for miRNA. *ciRS‐7*, the most well‐characterized circRNA, is stably and highly expressed in human and mouse brains. *ciRS‐7* has over 70 selective binding sites for miR‐7, which is associated with Argonaute (AGO) proteins, and can significantly repress miR‐7 activity, thereby increasing AGO protein levels,^35^, ^36^ suggesting a regulatory function for *ciRS‐7*. Moreover, the expression of *Fos*, an immediate early gene and a direct miR‐7 target, increases in *CDR1as*‐deficient brains and the animals display impaired sensorimotor gating, suggesting a possible link between circRNA and the behavioural phenotype.^30^ Particularly, circRNAs can be sponges for several miRNAs instead of containing binding sites for just one miRNA; for instance, circCCDC66 can bind to miR‐33b, miR‐93 and miR‐185.^37^

#### 2.4.2 Interaction with proteins

The circRNAs can also act as sponges for proteins, interacting with many different RBPs to influence protein functions. For example, circPABPN1 can bind to HuR, a type of RBP, preventing the binding between HuR and PABPN1 mRNA and subsequently reducing the latter's translation.^38^ Circ‐Foxo3, which is associated with cell cycle progression, is highly expressed in non‐cancer cells. Normally, cyclin‐dependent kinase 2 (CDK2) promotes cell cycle progression by interacting with cyclin A/E, whereas cyclin‐dependent kinase inhibitor 1 (p21) arrests this process. Ectopic expression of circ‐Foxo3, in the presence of CDK2 and p21, leads to ternary complex formation and CDK2 repression, resulting in a blockade of cell cycle progression.^39^ Another example is the MBL protein in *D. melanogaster*; MBL promotes the expression of circMbl, which binds to the protein itself and regulates parental gene expression. Excessive MBL protein decreases the expression of its linear mRNA by promoting circMbl expression, suggesting the presence of an autoregulatory circuit.^9^ Additionally, circ‐Amotl1 can bind to AKT1 and PDK1, resulting in phosphorylation of AKT1 and its translocation into the nucleus, a process involved in cardioprotection.^40^

#### 2.4.3 Templates for translation

Although circRNAs have been thought to be non‐coding, at least a subset of circRNAs can be translated into proteins under specific conditions.^41^, ^42^, ^43^ During the translation of linear mRNAs, a 7‐methylguanosine (m7G) cap at the 5′ end and a poly(A) tail at the 3′ end are essential elements. Due to the lack of both these structures, circRNAs are translated in a cap‐independent manner. One such mechanism is driven by N^6^‐methyladenosine (m6A) modification of the 5′ untranslated region. m6A can bind to eukaryotic initiation factor 3 (eIF3), facilitating recruitment of the 43S complex to initiate translation without the cap‐binding factor eIF4E.^44^ This process is promoted by methyltransferases (e.g. METTL3 and METTL14) and heat shock and restrained by demethylases (e.g. FTO).^42^ Another mechanism of circRNA translation relies on internal ribosome entry sites, which can accelerate binding between circRNAs and ribosomes or initiation factors, thereby promoting translation.^41^ CircRNAs containing repeated FLAG‐coding sequences can also be translated into proteins via rolling circle amplification.^45^ Through these different mechanisms, circRNA translation into functional proteins is possible. For example, FBXW7‐185aa, encoded by circ‐FBXW7, has potential prognostic implications in brain cancer,^43^ and the protein encoded by circ‐ZNF609 can specifically control myoblast proliferation.^41^

#### 2.4.4 Regulation of transcription

As mentioned above, EIciRNAs and ciRNAs mainly exist in the cell nucleus and some are involved in transcription regulation. EIciRNAs can interact with U1 small nuclear RNA to form a complex that binds Pol II at their parental gene promoters to upregulate gene expression.^7^ CircSCMH1 may interact with transcription factor methyl CpG binding protein 2 (MeCP2) to restrain its transcriptional activity. circMRPS35 can elicit the acetylation of H4K5 in the promoters by recruiting the histone acetyltransferase KAT7, and it can bind to *FOXO1* and *FOXO3a* promoters to activate transcription. In Arabidopsis, circRNAs derived from exon 6 of the SEP3 gene can bind to its cognate DNA locus to pause transcription.^46^ Ci‐ankrd52 has a stronger ability of R‐loop formation than that of its cognate pre‐mRNA, allowing the release of pre‐mRNA from R‐loops by ci‐ankrd52 replacement and followed by ciRNA removal via RNase H1 for efficient transcriptional elongation.^47^

## 3 circRNAs IN THE SKIN

The circRNAs are critical for physiological homeostasis and function through their modulation of gene expression in various tissues, including the skin. Indeed, the regulatory role of circRNAs in cutaneous wound healing has been well demonstrated. For instance, circ‐Amotl1 interacts with STAT3 to promote wound healing,^48^ whereas hsa_circ_0084443 is downregulated during normal wound healing and negatively regulates keratinocyte migration. Diabetic foot ulcers exhibit increased expression of hsa_circ_0084443, suggesting that its overexpression can contribute to the delayed wound healing.^49^ Knocking down circ‐PRKDC enhances keratinocyte migration and promotes skin wound healing; this is achieved through the miR‐31–FBN1 axis.^50^ Moreover, circRNAs can regulate keratinocyte differentiation, as inhibition of circZNF91, which contains 24 miR‐23b‐3p binding sites,^51^ reduces the expression of several proteins associated with keratinocyte differentiation.^52^ circRNAs have also been shown to regulate melanin production, and the evidence suggests that they are involved in the pigmentation and melanin‐related pathways.^53^ For instance, the interaction between lnRNA and circRNAs is associated with melanocyte development.^54^ *ciRS‐7*, which is highly expressed in melanocytes, triggers melanin production in melanocytes via regulation of the miR‐7–STAT3 and AKT–FGF2 paracrine axes, suggesting *ciRS‐7* as a regulator for the development of pigmented skin diseases.^55^ Moreover, circ_0091223 is involved in α‐MSH‐induced melanin production.^56^ Other regulatory roles of circRNAs in cutaneous functions include their involvement in keloid formation via the interaction between hsa_circ_0001320 and miR‐574‐5P,^57^, ^58^ skin tissue regeneration under mechanical tension,^59^ circCOL3A1–859267 inhibition of the UVA‐induced downregulation of type I collagen in skin^60^, ^61^; and the regulation of hair growth.^62^ Therefore, circRNAs can regulate multiple cutaneous functions.

## 4 circRNAs IN INFLAMMATORY DERMATOSES

### 4.1 Psoriasis

Psoriasis is a common inflammatory skin disease that presents with abnormal differentiation and excessive proliferation of epidermal cells as its main pathological features.^63^ Psoriasis has a great impact on the quality of life of patients.^64^ Although the pathogenic roles of keratinocytes, dendritic cells, T cells, neutrophils and mesenchymal stem cells in psoriasis are appreciated,^65^ the aetiology is extremely complex.

**FIGURE 2:**
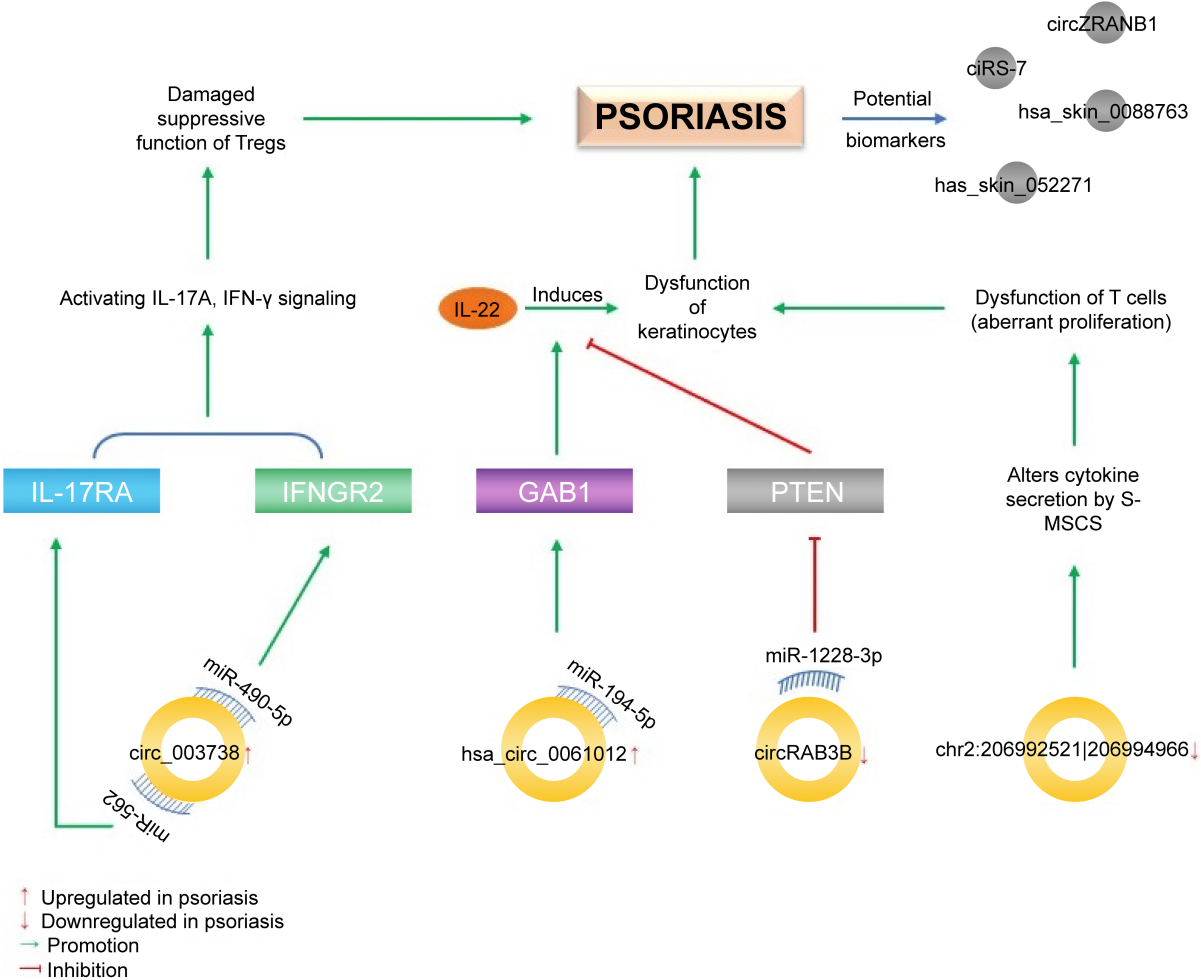
Regulatory network of circRNAs, their associated miRNAs and target genes in the pathogenesis of psoriasis. S‐MSCs, skin mesenchymal stem cells

Evidence has shown circRNA involvement in the pathogenesis and progression of psoriasis (Figure 2). To date, thousands of circRNAs that are differentially expressed in psoriasis, along with their related miRNAs and miRNA response elements, have been identified.^66^, ^67^, ^68^ Qiao et al.^66^ reported that hsa_circ_0061012 may participate in psoriasis via response to interleukin 4 (IL‐4), T cell selection and regulation of NF‐κB nuclear import. In particular, hsa_circ_0061012 has been implicated in the pathogenesis of psoriasis. Silencing hsa_circ_0061012 inhibits the proliferation, migration, and invasion of HaCaT cells induced by IL‐22, a key inflammatory cytokine involved in psoriasis development.^69^ MiR‐194‐5p inhibits this proliferation and migration of HaCaT cells partly by targeting *GAB1*, whereas hsa_circ_0061012 sponges miR‐194‐5p, increasing the expression of *GAB1* and subsequently enhancing HaCaT cell proliferation and migration.^70^ In contrast to hsa_circ_0061012, circRAB3B expression is decreased in psoriatic lesions. CircRAB3B sponges miR‐1228‐3p to upregulate PTEN, resulting in inhibition of psoriasis progression.^71^ Thus, both upregulated hsa_circ_0061012 and downregulated circRAB3B can contribute to psoriasis pathogenesis.

We have previously verified the downregulation of chr2:206992521|206994966 in skin mesenchymal stem cells (S‐MSCs) from psoriatic involved skin. According to a circRNA–miRNA–mRNA interaction network, some mRNAs associated with this circRNA are also downregulated in psoriatic S‐MSCs and are mainly involved in the MAPK and JAK–STAT signalling pathways.^67^, ^72^ The decrease in chr2:206992521|206994966 expression in psoriatic S‐MSCs hinders their ability to inhibit T cell proliferation, a feature of psoriasis. Likewise, knockdown of chr2:206992521|206994966 in S‐MSCs from normal skin induces a comparable decrease in the inhibitory ability of S‐MSCs on T cell proliferation and alters cytokine production, including that of IL‐6, IL‐11 and HGF, similar to the psoriatic S‐MSCs. These results suggest that chr2:206992521|206994966 can mediate the role of S‐MSCs in psoriasis.^72^

A study employing high‐throughput RNA sequencing demonstrated significantly downregulated expression of circRNA in the epidermis of psoriasis involved vs. uninvolved skin.^63^ In the peripheral venous blood, the levels of at least 205 circRNAs differ between patients with psoriasis and healthy controls. Kyoto Encyclopedia of Genes and Genomes (KEGG) enrichment analysis indicated that these differentially expressed circRNAs are mainly enriched in lipid metabolism, autoimmunity pathways, signal transduction and translation.^73^

It has been postulated that the compromised inhibitory function of regulatory T cells (Tregs) is linked to the etiopathogenesis and exacerbation of psoriasis.^74^ This is supported by several findings, including that circ_0003738 expression is significantly increased in psoriatic Tregs compared with normal controls and that circ_0003738 knockdown normalizes the inhibitory function of Tregs.^75^ Moreover, circ_0003738 can release the suppression of IL‐17 receptor A by binding to miR‐562, consequently promoting IL‐17A signalling in Tregs. Additionally, circ_0003738 serves as a sponge for miR‐490‐5p, relieving suppression of the target gene IFNGR2 and promoting IFN‐γ signalling in Tregs.^75^ These results suggest that circ_0003738 attenuates the inhibitory function of Tregs, which contributes to the onset of psoriasis.

Additionally, some circRNAs such as *ciRS‐7*, circZRANB1, hsa_skin_088763 and hsa_skin_052271 can serve as psoriasis biomarkers.^68^, ^76^ *CiRS‐7* and circZRANB1 expression can also aid in distinguishing atopic dermatitis (AD) from psoriasis.^76^

### 4.2 Lupus erythematosus

Lupus erythematosus is a diffuse connective tissue disease that affects many systems throughout the body. It is characterized by the presence of pathogenic autoantibodies and immune complexes that cause a loss of immune tolerance. Most patients exhibit cutaneous involvement.^77^ Systemic lupus erythematosus (SLE) usually presents with various clinical manifestations and multi‐systemic involvements, including skin and mucosal lesions, kidney damage, neuropsychiatric symptoms, haematological suppression, musculoskeletal damages and other non‐systemic symptoms.^78^ Its aetiology is highly complex and remains unclear, although the pathogenic role of T cells has been demonstrated.^79^ circRNAs are involved in SLE pathogenesis and development^80^, ^81^; for example, T cells from patients with SLE express reduced levels of hsa_circ_0045272, which has binding sites for hsa‐miR‐6127, resulting in downregulation of two mRNAs, NM_015177 (DTX4) and NM_003466 (PAX8).^82^ In activated Jurkat cells, knockdown of hsa_circ_0045272 significantly enhances IL‐2 production and promotes early apoptosis.^82^ Studies have also shown that in CD4+ T cells of SLE, reduced expression of hsa_circ_0012919, which can bind to miR‐125a‐3p, contributes to the methylation of *CD70*, *CD11a* and *MDA5*.^83^, ^84^ These findings suggest that circRNAs play a role in the abnormal autoimmune response of SLE.

In addition to T cells, altered levels of circRNA in the peripheral blood or peripheral blood mononuclear cells (PBMCs) have also been reported in patients with SLE. Endogenous circRNAs can form incomplete RNA duplexes (16–26 bp) that can suppress double‐stranded RNA‐dependent protein kinase (PKR), which is associated with innate immunity.^85^ PBMCs from patients with SLE show reduced circRNA expression and abnormal activation of PKR, suggesting a link between circRNAs and the abnormal autoimmune response in SLE.^85^ In addition, hsa_circ_0000479 expression is markedly increased in PBMCs from patients with SLE compared with healthy individuals. Bioinformatic analysis indicated that hsa_circ_0000479 regulates the Wnt signalling pathway and metabolic pathways, influencing SLE progression.^86^ Moreover, the levels of hsa_circRNA_001308 and hsa_circRNA_407176 are lower in both PBMCs and the plasma of patients with SLE than in healthy controls.^87^ In the peripheral blood of patients with SLE, expression levels of hsa_circ_0082688 and hsa_circ_0082689 are markedly increased while those of hsa_circ_0044235 and hsa_circ_0068367 are decreased in PBMCs.^88^, ^89^

Several circRNAs can be used as diagnostic markers for SLE. Indeed, the combination of anti‐dsDNA, hsa_circ_0082688 and hsa_circ_0082689 can distinguish patients with SLE from healthy controls with high sensitivity, specificity and accuracy.^88^ Similarly, hsa_circ_0000479 can be used to distinguish SLE from rheumatoid arthritis (RA).^86^ Correspondingly, the combination of anti‐dsDNA and hsa_circ_0000479‐ can differentiate SLE from ankylosing spondylitis (AS), RA and healthy controls.^90^

circRNA expression is also correlated with the severity of SLE. For example, circIBTK expression is negatively correlated with the Systemic Lupus Erythematosus Disease Activity Index (SLEDAI) score as well as the levels of complement C3 and anti‐dsDNA in patients with SLE.^91^ circIBTK can also bind to miR‐29b to regulate DNA demethylation and the AKT signalling pathway, suggesting both miR‐29 and circIBTK as potential biomarkers and therapeutic targets for SLE.^91^ Moreover, the levels of hsa_circ_0075699 and hsa_circ_0021372 in whole blood are correlated with complement C3 and C4 levels in children with SLE, while the level of hsa_circ_0057762 is positively correlated with the SLEDAI‐2000 score.^92^ In contrast, the expression of circPTPN22, has_circ_0049220, and hsa_circ_0049224 is inversely correlated with the SLEDAI score.^93^, ^94^ An association between hsa_circ_0000479 and hsa_circ_0082688 with the levels of complement C3 and anti‐dsDNA, respectively, has also been reported.

In addition to SLE, the lesion of discoid lupus erythematosus significantly and differentially expresses at least 161 circRNAs that are associated with the immune response, inflammatory reaction, and T cell co‐stimulation, as evidenced by gene ontology (GO) analysis.^95^

### 4.3 Atopic dermatitis

As another common chronic inflammatory dermatosis, AD is linked to genetic predisposition, immune system disorder and epidermal barrier disruption.^96^ Although the exact role of circRNAs in AD is not fully understood, their expression is significantly altered in AD lesions compared with non‐lesional and healthy skin. For example, circRHOBTB3, circDEF6, circCCDC7, circSWT1 and circDEGS1 are significantly downregulated, whereas circDDX21 and circTNFRSF21 are significantly upregulated in AD skin. Overall, circRNAs are globally downregulated in lesional skin compared with non‐lesional skin, and their expression is negatively correlated with the Scoring Atopic Dermatitis Index. Interestingly, circRNA expression in non‐lesional skin from male patients varied more than that of female patients compared with healthy skin samples, with male patients showing a higher mean severity score.^76^

### 4.4 Lichen planus

Lichen planus is an inflammatory mucocutaneous condition that affects the oral and genital mucous membranes in addition to the skin and typically develops in middle‐aged adults, especially females.^97^, ^98^ Although lichen planus is considered a T cell‐mediated autoimmune disease,^99^ its pathogenesis remains nebulous. A recent study identified abnormal expression of 135 circRNAs in oral lichen planus (OLP); among them, chr6:31238920–31324013‐, whose host gene is HLA‐C (a regulator of the human immune system), is significantly highly expressed in OLP.^100^ High expression of HLA‐C on the cell surface can aggravate cytotoxic T cell responses.^101^ Moreover, hsa_circ_0006867, which is derived from LRBA, is downregulated in OLP.^100^ LRBA is involved in the secretion and membrane deposition of immune effector molecules. A lack of LRBA can cause defects in Tregs, resulting in immune disorders.^102^ These results suggest that some circRNAs play a significant role in the pathogenesis of OLP.

### 4.5 Severe acne

Acne, characterized by chronic inflammation of the pilosebaceous unit,^103^ is estimated to affect 85% of individuals between the ages of 12 and 25,^104^ making it the eighth most prevalent disease in the world.^105^ Because acne negatively affects both the physical and mental health of patients, its pathogenesis has attracted attention. *Propionibacterium acnes*, androgen, growth factors and inflammatory factors have all been proposed to play important roles in acne development.^104^ Some circRNAs may also be involved in the onset of severe acne since lesional and non‐lesional skin from patients with severe acne differentially expresses at least 538 circRNAs, including 271 upregulated and 267 downregulated circRNAs.^106^ GO analyses and KEGG pathway enrichment showed that these circRNAs are related to metabolism, immune responses and inflammation. qPCR analysis also demonstrated significantly decreased expression of five circRNAs in severe acne. It is predicted that these five circRNAs regulate target gene expression by interacting with 213 miRNAs, highlighting circRNAs as potential biomarkers or drug targets of severe acne.^106^

The role of circRNAs in inflammatory skin diseases other than psoriasis is shown in Table 1.

**TABLE 1:** circRNA in other inflammatory dermatoses

Diseases	CircRNA	Expression	Targets	Functions	Reference
Lupus erythematosus	hsa_circ_0045272	Down in T cells	hsa‐miR‐6127 NM_003466 (PAX8) NM_015177 (DTX4)	Increasing IL‐2 production of activated Jurkat cells and upregulating early apoptosis.	82
	hsa_circ_0012919	Down in CD4^+^ T cells	miR‐125a‐3p	Contributing to DNA methylation of CD11a, CD70 and MDA5 in CD4^+^ T cells.	83, 84
	hsa_circ_0000479	Up in PBMCs		Modulating metabolic pathways and the Wnt signalling pathway to regulate SLE progression. Distinguishing SLE from RA, AS and healthy people.	86
	hsa_circRNA_407176 hsa_circRNA_00130 hsa_circ_0068367	Down in PBMCs			87, 89
	hsa_circ_0044235	Down in PBMCs	hsa‐miRNA‐892a		89
	hsa_circ_0082688 hsa_circ_0082689	Up in peripheral blood		Hsa_circ_0082688‐hsa_circ_0082689 + anti‐dsDNA could effectively discriminate the SLE group from the control.	88
	circIBTK	Down in PBMCs	miR‐29b	Regulating DNA demethylation and the AKT signalling pathway. Correlated with SLEDAI score, (ds)DNA and complement C3 level.	91
	hsa_circ_0021372	Down in whole blood		Associated with C3 and C4 levels.	92
	hsa_circ_0075699	Up in whole blood		Associated with C3 and C4 levels.	92
	hsa_circ_0057762	Down in whole blood		Associated with the SLEDAI‐2 K score.	92
	circPTPN22 hsa_circ_0049224 has_circ_0049220	Down in PBMCs		Negative associated with SLEDAI score.	93, 94
Atopic dermatitis	circRHOBTB3 circDEGS1 circDEF6 circSWT1 circCCDC7	Down in the lesional skin		Negatively correlated with SCORAD index.	76
	circTNFRSF21 circDDX21	Up in the lesional skin			76
Lichen planus	chr6:31238920–31324013	Up in oral mucosal tissues	HLA‐C	Leading to aggravating cytotoxic T cell responses.	100
	hsa_circ_0006867	Down in oral mucosal tissues	LRBA	Related to defects in regulatory T cells and causing immune disorders.	100
Severe acne	circRNA_0084927	Down in lesional skin		Related to inflammatory, metabolism and immune responses.	106
	circRNA_0001073				
	circRNA_0005941				
	circRNA_0086376				
	circRNA_0018168				

Abbreviations: AS, ankylosing spondylitis; circRNA, circular RNA; dsDNA, double‐stranded DNA; IL‐2, interleukin 2; PBMCs, peripheral blood  mononuclear cells; RA, rheumatoid arthritis; SCORAD, Scoring Atopic Dermatitis; SLE, systemic lupus erythematosus; SLEDAI, Systemic Lupus Erythematosus Disease Activity Index.

## 5 CONCLUSION AND FUTURE PERSPECTIVES

circRNAs are an emerging research hotspot in the medical field owing to their great potential in the occurrence, development, diagnosis and treatment of several diseases. They also show potential as biomarkers for the diagnosis and assessment of disease severity or as effective therapeutic targets for many inflammatory dermatoses, including psoriasis, lupus erythematosus, AD, lichen planus and severe acne.

Although circRNAs are known to have multiple functions, such as miRNA sponge activity,^30^, ^31^, ^32^, ^33^, ^34^, ^35^, ^36^, ^37^ protein interaction,^13^, ^38^, ^39^, ^40^ translation templates^41^, ^42^, ^43^ and transcriptional regulation, studies on circRNAs in inflammatory dermatoses have mainly focussed on changes in their expression and their function as miRNA sponges. Moreover, data on the specific mechanism by which circRNAs contribute to the pathogenesis of certain diseases remain limited,^70^ along with data regarding the specific signalling pathways or molecular mechanism by which circRNAs act. Furthermore, it is not clear whether the changes in circRNA expression are the cause or symptom of the disease. Thus, studies on the specific mechanisms of circRNAs in inflammatory skin diseases are crucial for elucidating the pathomechanisms and developing circRNA‐based therapeutics. Importantly, large sample sizes are required to validate the significance of circRNA expression in the diagnosis and assessment of disease severity as well their potential as therapeutic targets.

## AUTHOR CONTRIBUTIONS

**Ruifeng Liu:** Writing – original draft (lead); writing – review and editing (lead). **Luyao Zhang:** Writing – original draft (equal); writing – review and editing (equal). **Xincheng Zhao:** Investigation (equal); resources (equal). **Jia Liu:** Investigation (equal); resources (equal). **Wenjuan Chang:** Investigation (equal); resources (equal). **Ling Zhou:** Investigation (equal); resources (equal). **Kaiming Zhang:** Writing – review and editing (equal).

## CONFLICT OF INTEREST

The authors declare that they have no conflict of interest.

## Data Availability

The data that support the findings of this study are available from the corresponding author upon reasonable request.

## References

[1] Li F , Yang Q , He AT , Yang BB . Circular RNAs in cancer: limitations in functional studies and diagnostic potential. Semin Cancer Biol. 2021;75:49‐61. 33035655 10.1016/j.semcancer.2020.10.002

[2] Bei Y , Yang T , Wang L , et al. Circular RNAs as potential Theranostics in the cardiovascular system. Mol Ther Nucleic Acids. 2018;13:407‐418. 30368217 10.1016/j.omtn.2018.09.022 PMC6205062

[3] Yan L , Chen YG . Circular RNAs in immune response and viral infection. Trends Biochem Sci. 2020;45(12):1022‐1034. 32900574 10.1016/j.tibs.2020.08.006 PMC7642119

[4] Diener TO . Potato spindle tuber “virus”. IV. A replicating, low molecular weight RNA. Virology. 1971;45(2):411‐428. 5095900 10.1016/0042-6822(71)90342-4

[5] Weiner AJ , Choo QL , Wang KS , et al. A single antigenomic open reading frame of the hepatitis delta virus encodes the epitope(s) of both hepatitis delta antigen polypeptides p24 delta and p27 delta. J Virol. 1988;62(2):594‐599. 2447291 10.1128/jvi.62.2.594-599.1988 PMC250573

[6] Salzman J , Chen RE , Olsen MN , Wang PL , Brown PO . Cell‐type specific features of circular RNA expression. PLoS Genet. 2013;9(9):e1003777. 24039610 10.1371/journal.pgen.1003777 PMC3764148

[7] Li Z , Huang C , Bao C , et al. Exon‐intron circular RNAs regulate transcription in the nucleus. Nat Struct Mol Biol. 2015;22(3):256‐264. 25664725 10.1038/nsmb.2959

[8] Zhang Y , Zhang XO , Chen T , et al. Circular intronic long noncoding RNAs. Mol Cell. 2013;51(6):792‐806. 24035497 10.1016/j.molcel.2013.08.017

[9] Memczak S , Jens M , Elefsinioti A , et al. Circular RNAs are a large class of animal RNAs with regulatory potency. Nature. 2013;495(7441):333‐338. 23446348 10.1038/nature11928

[10] Hu Q , Zhou T . EIciRNA‐mediated gene expression: tunability and bimodality. FEBS Lett. 2018;592(20):3460‐3471. 30223292 10.1002/1873-3468.13253

[11] Liang D , Wilusz JE . Short intronic repeat sequences facilitate circular RNA production. Genes Dev. 2014;28(20):2233‐2247. 25281217 10.1101/gad.251926.114 PMC4201285

[12] Yoshimoto R , Rahimi K , Hansen TB , Kjems J , Mayeda A . Biosynthesis of circular RNA ciRS‐7/CDR1as is mediated by mammalian‐wide interspersed repeats. iScience. 2020;23(7):101345. 32683316 10.1016/j.isci.2020.101345 PMC7371899

[13] Ashwal‐Fluss R , Meyer M , Pamudurti NR , et al. circRNA biogenesis competes with pre‐mRNA splicing. Mol Cell. 2014;56(1):55‐66. 25242144 10.1016/j.molcel.2014.08.019

[14] Fei T , Chen Y , Xiao T , et al. Genome‐wide CRISPR screen identifies HNRNPL as a prostate cancer dependency regulating RNA splicing. Proc Natl Acad Sci U S A. 2017;114(26):E5207‐E5215. 28611215 10.1073/pnas.1617467114 PMC5495225

[15] Conn SJ , Pillman KA , Toubia J , et al. The RNA binding protein quaking regulates formation of circRNAs. Cell. 2015;160(6):1125‐1134. 25768908 10.1016/j.cell.2015.02.014

[16] Errichelli L , Dini Modigliani S , Laneve P , et al. FUS affects circular RNA expression in murine embryonic stem cell‐derived motor neurons. Nat Commun. 2017;8:14741. 28358055 10.1038/ncomms14741 PMC5379105

[17] Li X , Liu CX , Xue W , et al. Coordinated circRNA biogenesis and function with NF90/NF110 in viral infection. Mol Cell. 2017;67(2):214‐227.e7. 28625552 10.1016/j.molcel.2017.05.023

[18] Zhang Y , Xue W , Li X , et al. The biogenesis of nascent circular RNAs. Cell Rep. 2016;15(3):611‐624. 27068474 10.1016/j.celrep.2016.03.058

[19] Liang D , Tatomer DC , Luo Z , et al. The output of protein‐coding genes shifts to circular RNAs when the pre‐mRNA processing machinery is limiting. Mol Cell. 2017;68(5):940‐954.e3. 29174924 10.1016/j.molcel.2017.10.034 PMC5728686

[20] Enuka Y , Lauriola M , Feldman ME , Sas‐Chen A , Ulitsky I , Yarden Y . Circular RNAs are long‐lived and display only minimal early alterations in response to a growth factor. Nucleic Acids Res. 2016;44(3):1370‐1383. 26657629 10.1093/nar/gkv1367 PMC4756822

[21] Suzuki H , Tsukahara T . A view of pre‐mRNA splicing from RNase R resistant RNAs. Int J Mol Sci. 2014;15(6):9331‐9342. 24865493 10.3390/ijms15069331 PMC4100097

[22] Jeck WR , Sharpless NE . Detecting and characterizing circular RNAs. Nat Biotechnol. 2014;32(5):453‐461. 24811520 10.1038/nbt.2890 PMC4121655

[23] Jeck WR , Sorrentino JA , Wang K , et al. Circular RNAs are abundant, conserved, and associated with ALU repeats. RNA. 2013;19(2):141‐157. 23249747 10.1261/rna.035667.112 PMC3543092

[24] Zhang XO , Wang HB , Zhang Y , Lu X , Chen LL , Yang L . Complementary sequence‐mediated exon circularization. Cell. 2014;159(1):134‐147. 25242744 10.1016/j.cell.2014.09.001

[25] Rybak‐Wolf A , Stottmeister C , Glažar P , et al. Circular RNAs in the mammalian brain are highly abundant, conserved, and dynamically expressed. Mol Cell. 2015;58(5):870‐885. 25921068 10.1016/j.molcel.2015.03.027

[26] You X , Vlatkovic I , Babic A , et al. Neural circular RNAs are derived from synaptic genes and regulated by development and plasticity. Nat Neurosci. 2015;18(4):603‐610. 25714049 10.1038/nn.3975 PMC4376664

[27] Gruner H , Cortés‐López M , Cooper DA , Bauer M , Miura P . CircRNA accumulation in the aging mouse brain. Sci Rep. 2016;6:38907. 27958329 10.1038/srep38907 PMC5153657

[28] Dong R , Ma XK , Chen LL , Yang L . Increased complexity of circRNA expression during species evolution. RNA Biol. 2017;14(8):1064‐1074. 27982734 10.1080/15476286.2016.1269999 PMC5680680

[29] Salmena L , Poliseno L , Tay Y , Kats L , Pandolfi PP . A ceRNA hypothesis: the Rosetta stone of a hidden RNA language? Cell. 2011;146(3):353‐358. 21802130 10.1016/j.cell.2011.07.014 PMC3235919

[30] Piwecka M , Glažar P , Hernandez‐Miranda LR , et al. Loss of a mammalian circular RNA locus causes miRNA deregulation and affects brain function. Science. 2017;357(6357):eaam8526. 28798046 10.1126/science.aam8526

[31] Zheng Q , Bao C , Guo W , et al. Circular RNA profiling reveals an abundant circHIPK3 that regulates cell growth by sponging multiple miRNAs. Nat Commun. 2016;7:11215. 27050392 10.1038/ncomms11215 PMC4823868

[32] Peng F , Gong W , Li S , et al. circRNA_010383 acts as a sponge for miR‐135a, and its downregulated expression contributes to renal fibrosis in diabetic nephropathy. Diabetes. 2021;70(2):603‐615. 33472945 10.2337/db20-0203

[33] Peng L , Chen G , Zhu Z , et al. Circular RNA ZNF609 functions as a competitive endogenous RNA to regulate AKT3 expression by sponging miR‐150‐5p in Hirschsprung's disease. Oncotarget. 2017;8(1):808‐818. 27903978 10.18632/oncotarget.13656 PMC5352198

[34] Gao Y , Liu J , Huan J , Che F . Downregulation of circular RNA hsa_circ_0000735 boosts prostate cancer sensitivity to docetaxel via sponging miR‐7. Cancer Cell Int. 2020;20:334. 32714093 10.1186/s12935-020-01421-6 PMC7376840

[35] Weng W , Wei Q , Toden S , et al. Circular RNA ciRS‐7—a promising prognostic biomarker and a potential therapeutic target in colorectal cancer. Clin Cancer Res. 2017;23(14):3918‐3928. 28174233 10.1158/1078-0432.CCR-16-2541 PMC5511556

[36] Yu L , Gong X , Sun L , Zhou Q , Lu B , Zhu L . The circular RNA Cdr1as act as an oncogene in hepatocellular carcinoma through targeting miR‐7 expression. PLoS One. 2016;11(7):e0158347. 27391479 10.1371/journal.pone.0158347 PMC4938625

[37] Hsiao KY , Lin YC , Gupta SK , et al. Noncoding effects of circular RNA CCDC66 promote colon cancer growth and metastasis. Cancer Res. 2017;77(9):2339‐2350. 28249903 10.1158/0008-5472.CAN-16-1883 PMC5910173

[38] Abdelmohsen K , Panda AC , Munk R , et al. Identification of HuR target circular RNAs uncovers suppression of PABPN1 translation by CircPABPN1. RNA Biol. 2017;14(3):361‐369. 28080204 10.1080/15476286.2017.1279788 PMC5367248

[39] Du WW , Yang W , Liu E , Yang Z , Dhaliwal P , Yang BB . Foxo3 circular RNA retards cell cycle progression via forming ternary complexes with p21 and CDK2. Nucleic Acids Res. 2016;44(6):2846‐2858. 26861625 10.1093/nar/gkw027 PMC4824104

[40] Zeng Y , Du WW , Wu Y , et al. A circular RNA binds to and activates AKT phosphorylation and nuclear localization reducing apoptosis and enhancing cardiac repair. Theranostics. 2017;7(16):3842‐3855. 29109781 10.7150/thno.19764 PMC5667408

[41] Legnini I , Di Timoteo G , Rossi F , et al. Circ‐ZNF609 is a circular RNA that can be translated and functions in myogenesis. Mol Cell. 2017;66(1):22‐37.e9. 28344082 10.1016/j.molcel.2017.02.017 PMC5387670

[42] Yang Y , Fan X , Mao M , et al. Extensive translation of circular RNAs driven by N (6)‐methyladenosine. Cell Res. 2017;27(5):626‐641. 28281539 10.1038/cr.2017.31 PMC5520850

[43] Yang Y , Gao X , Zhang M , et al. Novel role of FBXW7 circular RNA in repressing glioma tumorigenesis. J Natl Cancer Inst. 2018;110(3):304‐315. 10.1093/jnci/djx166 PMC6019044 28903484

[44] Meyer KD , Patil DP , Zhou J , et al. 5’ UTR m(6)a promotes cap‐independent translation. Cell. 2015;163(4):999‐1010. 26593424 10.1016/j.cell.2015.10.012 PMC4695625

[45] Abe N , Matsumoto K , Nishihara M , et al. Rolling circle translation of circular RNA in living human cells. Sci Rep. 2015;5:16435. 26553571 10.1038/srep16435 PMC4639774

[46] Wang X , Ma R , Zhang X , et al. Crosstalk between N6‐methyladenosine modification and circular RNAs: current understanding and future directions. Mol Cancer. 2021;20(1):121. 34560891 10.1186/s12943-021-01415-6 PMC8461955

[47] Li X , Zhang JL , Lei YN , et al. Linking circular intronic RNA degradation and function in transcription by RNase H1. Sci China Life Sci. 2021 Nov;64(11):1795‐1809. 34453665 10.1007/s11427-021-1993-6

[48] Yang ZG , Awan FM , Du WW , et al. The circular RNA interacts with STAT3, increasing its nuclear translocation and wound repair by modulating Dnmt3a and miR‐17 function. Mol Ther. 2017;25(9):2062‐2074. 28676341 10.1016/j.ymthe.2017.05.022 PMC5589065

[49] Wang A , Toma MA , Ma J , et al. Circular RNA hsa_circ_0084443 is upregulated in diabetic foot ulcer and modulates keratinocyte migration and proliferation. Adv Wound Care. 2020;9(4):145‐160. 10.1089/wound.2019.0956 PMC7047102 32117579

[50] Han D , Liu W , Li G , Liu L . Circ_PRKDC knockdown promotes skin wound healing by enhancing keratinocyte migration via miR‐31/FBN1 axis. J Mol Histol. 2021;52(4):681‐691. 34143322 10.1007/s10735-021-09996-8

[51] Kristensen LS , Okholm TLH , Venø MT , Kjems J . Circular RNAs are abundantly expressed and upregulated during human epidermal stem cell differentiation. RNA Biol. 2018;15(2):280‐291. 29283313 10.1080/15476286.2017.1409931 PMC5798954

[52] Barbollat‐Boutrand L , Joly‐Tonetti N , Dos Santos M , et al. MicroRNA‐23b‐3p regulates human keratinocyte differentiation through repression of TGIF1 and activation of the TGF‐β‐SMAD2 signalling pathway. Exp Dermatol. 2017;26(1):51‐57. 27306475 10.1111/exd.13119

[53] Zhu Z , Li Y , Liu W , et al. Comprehensive circRNA expression profile and construction of circRNA‐associated ceRNA network in fur skin. Exp Dermatol. 2018;27(3):251‐257. 29377327 10.1111/exd.13502

[54] Zhu Z , Ma Y , Li Y , et al. The comprehensive detection of miRNA, lncRNA, and circRNA in regulation of mouse melanocyte and skin development. Biol Res. 2020;53(1):4. 32014065 10.1186/s40659-020-0272-1 PMC6998077

[55] Ouyang Y , Chen J , Jiang L , et al. UVB‐induced ciRS‐7 activates melanogenesis by paracrine effects. DNA Cell Biol. 2021;40(3):523‐531. 33687273 10.1089/dna.2020.5489

[56] Jiang L , Huang J , Hu Y , et al. Identification of the ceRNA networks in α‐MSH‐induced melanogenesis of melanocytes. Aging. 2020;13(2):2700‐2726. 33318297 10.18632/aging.202320 PMC7880406

[57] Wang J , Wu H , Xiao Z , Dong X . Expression profiles of lncRNAs and circRNAs in keloid. Plast Reconstr Surg Glob Open. 2019;7(6):e2265. 31624676 10.1097/GOX.0000000000002265 PMC6635192

[58] Zhang J , Liu N , Wu X , Wu P , Song N , Ma J . Identification of differentially expressed circular RNAs in keloid and normal skin tissue by high‐throughput sequencing. Dermatol Ther. 2021;34(2):e14745. 33405341 10.1111/dth.14745

[59] Liu Z , Liu W , Fan J , et al. Effect of mechanical tension on the circRNA expression profile of human skin tissue. J Craniofac Surg. 2019;30(5):e474‐e477. 31299820 10.1097/SCS.0000000000005592

[60] Peng Y , Song X , Zheng Y , Cheng H , Lai W . circCOL3A1–859267 regulates type I collagen expression by sponging miR‐29c in human dermal fibroblasts. Eur J Dermatol. 2018;28(5):613‐620. 30378537 10.1684/ejd.2018.3397

[61] Peng Y , Song X , Zheng Y , Wang X , Lai W . Circular RNA profiling reveals that circCOL3A1–859267 regulate type I collagen expression in photoaged human dermal fibroblasts. Biochem Biophys Res Commun. 2017;486(2):277‐284. 28286269 10.1016/j.bbrc.2017.03.028

[62] Zhao R , Liu N , Han F , et al. Identification and characterization of circRNAs in the skin during wool follicle development in Aohan fine wool sheep. BMC Genomics. 2020;21(1):187. 32111155 10.1186/s12864-020-6599-8 PMC7048093

[63] Moldovan LI , Hansen TB , Venø MT , et al. High‐throughput RNA sequencing from paired lesional‐ and non‐lesional skin reveals major alterations in the psoriasis circRNAome. BMC Med Genomics. 2019;12(1):174. 31775754 10.1186/s12920-019-0616-2 PMC6882360

[64] Kim WB , Jerome D , Yeung J . Diagnosis and management of psoriasis. Can Fam Physician. 2017;63(4):278‐285. 28404701 PMC5389757

[65] Takahashi T , Yamasaki K . Psoriasis and antimicrobial peptides. Int J Mol Sci. 2020;21(18):6791. 10.3390/ijms21186791 PMC7555190 32947991

[66] Qiao M , Ding J , Yan J , Li R , Jiao J , Sun Q . Circular RNA expression profile and analysis of their potential function in psoriasis. Cell Physiol Biochem. 2018;50(1):15‐27. 30278433 10.1159/000493952

[67] Liu R , Wang Q , Chang W , Zhou L , Li J , Zhang K . Characterisation of the circular RNA landscape in mesenchymal stem cells from psoriatic skin lesions. Eur J Dermatol. 2019;29(1):29‐38. 30827946 10.1684/ejd.2018.3483

[68] Liu X , Frost J , Bowcock A , Zhang W . Canonical and interior circular RNAs function as competing endogenous RNAs in psoriatic skin. Int J Mol Sci. 2021;22(10):5182. 34068434 10.3390/ijms22105182 PMC8153647

[69] Hao JQ . Targeting interleukin‐22 in psoriasis. Inflammation. 2014;37(1):94‐99. 23978911 10.1007/s10753-013-9715-y

[70] He Q , Liu N , Hu F , et al. Circ_0061012 contributes to IL‐22‐induced proliferation, migration and invasion in keratinocytes through miR‐194‐5p/GAB1 axis in psoriasis. Biosci Rep. 2021;41(1):BSR20203130. 33393621 10.1042/BSR20203130 PMC7809556

[71] Lu J , Xu X , Li Y , Yu N , Ding Y , Shi Y . CircRAB3B suppresses proliferation, motility, cell cycle progression and promotes the apoptosis of IL‐22‐induced keratinocytes depending on the regulation of miR‐1228‐3p/PTEN axis in psoriasis. Autoimmunity. 2021;54(5):303‐312. 34096408 10.1080/08916934.2021.1934825

[72] Liu R , Chang W , Li J , et al. Mesenchymal stem cells in psoriatic lesions affect the skin microenvironment through circular RNA. Exp Dermatol. 2019;28(3):292‐299. 30664808 10.1111/exd.13890

[73] Chen Z , Wang Y , Zhao J , et al. A study on the pathogenesis of blood‐heat psoriasis with transcriptome analysis. Ann Transl Med. 2020;8(22):1523. 33313268 10.21037/atm-20-7137 PMC7729302

[74] Nussbaum L , Chen YL , Ogg GS . Role of regulatory T cells in psoriasis pathogenesis and treatment. Br J Dermatol. 2021;184(1):14‐24. 32628773 10.1111/bjd.19380

[75] Yang L , Zhang C , Bai X , Xiao C , Dang E , Wang G . hsa_circ_0003738 inhibits the suppressive function of Tregs by targeting miR‐562/IL‐17A and miR‐490‐5p/IFN‐γ signaling pathway. Mol Ther Nucleic Acids. 2020;21:1111‐1119. 32871353 10.1016/j.omtn.2020.08.001 PMC7475646

[76] Moldovan LI , Tsoi LC , Ranjitha U , et al. Characterization of circular RNA transcriptomes in psoriasis and atopic dermatitis reveals disease‐specific expression profiles. Exp Dermatol. 2021;30(8):1187‐1196. 33113213 10.1111/exd.14227

[77] Kuhn A , Landmann A . The classification and diagnosis of cutaneous lupus erythematosus. J Autoimmun. 2014;48–49:14‐19. 10.1016/j.jaut.2014.01.021 24486120

[78] Zheng F , Yu X , Tang D , et al. The identification of circular RNAs from peripheral blood mononuclear cells in systemic lupus erythematosus. BMC Med Genomics. 2021;14(1):70. 33750387 10.1186/s12920-021-00919-w PMC7941743

[79] Zhang J , Liu Y , Shi G . The circRNA‐miRNA‐mRNA regulatory network in systemic lupus erythematosus. Clin Rheumatol. 2021;40(1):331‐339. 32533339 10.1007/s10067-020-05212-2

[80] Wang X , Ma R , Shi W , Wu Z , Shi Y . Emerging roles of circular RNAs in systemic lupus erythematosus. Mol Ther Nucleic Acids. 2021;24:212‐222. 33767917 10.1016/j.omtn.2021.02.028 PMC7973136

[81] Liu H , Zou Y , Chen C , Tang Y , Guo J . Current understanding of circular RNAs in systemic lupus erythematosus. Front Immunol. 2021;12:628872. 33717154 10.3389/fimmu.2021.628872 PMC7946848

[82] Li LJ , Zhu ZW , Zhao W , et al. Circular RNA expression profile and potential function of hsa_circ_0045272 in systemic lupus erythematosus. Immunology. 2018;155(1):137‐149. 29700819 10.1111/imm.12940 PMC6099170

[83] Zhang C , Wang X , Chen Y , Wu Z , Zhang C , Shi W . The down‐regulation of hsa_circ_0012919, the sponge for miR‐125a‐3p, contributes to DNA methylation of CD11a and CD70 in CD4(+) T cells of systemic lupus erythematous. Clin Sci. 2018;132(21):2285‐2298. 10.1042/CS20180403 30237316

[84] Zhang C , Zhang C , Ji J , Xiong X , Lu Y . Hsa_circ_0012919 regulates expression of MDA5 by miR‐125a‐3p in CD4+ T cells of systemic lupus erythematous. Lupus. 2020;29(7):727‐734. 32321346 10.1177/0961203320920706

[85] Liu CX , Li X , Nan F , et al. Structure and degradation of circular RNAs regulate PKR activation in innate immunity. Cell. 2019;177(4):865‐880.e21. 31031002 10.1016/j.cell.2019.03.046

[86] Guo G , Wang H , Ye L , et al. Hsa_circ_0000479 as a novel diagnostic biomarker of systemic lupus erythematosus. Front Immunol. 2019;10:2281. 31608065 10.3389/fimmu.2019.02281 PMC6771011

[87] Zhang MY , Wang JB , Zhu ZW , et al. Differentially expressed circular RNAs in systemic lupus erythematosus and their clinical significance. Biomed Pharmacother. 2018;107:1720‐1727. 30257390 10.1016/j.biopha.2018.08.161

[88] Luo Q , Li X , Fu B , et al. Expression profile and diagnostic value of circRNAs in peripheral blood from patients with systemic lupus erythematosus. Mol Med Rep. 2021;23(1):1. 10.3892/mmr.2020.11639 PMC7673322 33169172

[89] Luo Q , Zhang L , Li X , et al. Identification of circular RNAs hsa_circ_0044235 and hsa_circ_0068367 as novel biomarkers for systemic lupus erythematosus. Int J Mol Med. 2019;44(4):1462‐1472. 31432107 10.3892/ijmm.2019.4302 PMC6713423

[90] Luo Q , Zhang L , Fang L , et al. Circular RNAs hsa_circ_0000479 in peripheral blood mononuclear cells as novel biomarkers for systemic lupus erythematosus. Autoimmunity. 2020;53(3):167‐176. 32093518 10.1080/08916934.2020.1728529

[91] Wang X , Zhang C , Wu Z , Chen Y , Shi W . CircIBTK inhibits DNA demethylation and activation of AKT signaling pathway via miR‐29b in peripheral blood mononuclear cells in systemic lupus erythematosus. Arthritis Res Ther. 2018;20(1):118. 29884225 10.1186/s13075-018-1618-8 PMC5993996

[92] Li S , Zhang J , Tan X , et al. Microarray expression profile of circular RNAs and mRNAs in children with systemic lupus erythematosus. Clin Rheumatol. 2019;38(5):1339‐1350. 30628013 10.1007/s10067-018-4392-8

[93] Miao Q , Zhong Z , Jiang Z , et al. RNA‐seq of circular RNAs identified circPTPN22 as a potential new activity indicator in systemic lupus erythematosus. Lupus. 2019;28(4):520‐528. 30871426 10.1177/0961203319830493

[94] Zhang C , Huang J , Chen Y , Shi W . Low expression and clinical value of hsa_circ_0049224 and has_circ_0049220 in systemic lupus erythematous patients. Med Sci Monit. 2018;24:1930‐1935. 29606700 10.12659/MSM.906507 PMC5898388

[95] Xuan J , Xiong Y , Shi L , Aramini B , Wang H . Do lncRNAs and circRNAs expression profiles influence discoid lupus erythematosus progression? a comprehensive analysis. Ann Transl Med. 2019;7(23):728. 32042744 10.21037/atm.2019.12.10 PMC6990042

[96] Kim J , Kim BE , Leung DYM . Pathophysiology of atopic dermatitis: clinical implications. Allergy Asthma Proc. 2019;40(2):84‐92. 30819278 10.2500/aap.2019.40.4202 PMC6399565

[97] Mauskar M . Erosive Lichen Planus. Obstet Gynecol Clin North Am. 2017;44(3):407‐420. 28778640 10.1016/j.ogc.2017.04.004

[98] Parlatescu I , Tovaru M , Nicolae CL , Sfeatcu R , Didilescu AC . Oral health‐related quality of life in different clinical forms of oral lichen planus. Clin Oral Investig. 2020;24(1):301‐308. 10.1007/s00784-019-02951-8 31098713

[99] Wang H , Zhang D , Han Q , et al. Role of distinct CD4(+) T helper subset in pathogenesis of oral lichen planus. J Oral Pathol Med. 2016;45(6):385‐393. 26693958 10.1111/jop.12405

[100] Song Y , Xu S , Shao Y , Ge S , Zhou H . Expression profile of circular RNAs in oral lichen planus. Ann Palliat Med. 2021;10(5):5205‐5217. 34044570 10.21037/apm-20-2253

[101] van den Bogaard EH , Tijssen HJ , Rodijk‐Olthuis D , et al. Cell surface expression of HLA‐Cw6 by human epidermal keratinocytes: positive regulation by cytokines, lack of correlation to a variant upstream of HLA‐C. J Invest Dermatol. 2016;136(9):1903‐1906. 27297019 10.1016/j.jid.2016.05.112

[102] Tesch VK , Abolhassani H , Shadur B , et al. Long‐term outcome of LRBA deficiency in 76 patients after various treatment modalities as evaluated by the immune deficiency and dysregulation activity (IDDA) score. J Allergy Clin Immunol. 2020;145(5):1452‐1463. 31887391 10.1016/j.jaci.2019.12.896

[103] Kircik LH . Advances in the understanding of the pathogenesis of inflammatory acne. J Drugs Dermatol. 2016;15(1 Suppl 1):s7‐s10. 26741394

[104] Gollnick HP , Dreno B . Pathophysiology and management of acne. J Eur Acad Dermatol Venereol. 2015;29(Suppl 4):1‐2. 10.1111/jdv.13182 26059727

[105] Tan JK , Bhate K . A global perspective on the epidemiology of acne. Br J Dermatol. 2015;172(Suppl 1):3‐12. 10.1111/bjd.13462 25597339

[106] Liang J , Wu X , Sun S , et al. Circular RNA expression profile analysis of severe acne by RNA‐seq and bioinformatics. J Eur Acad Dermatol Venereol. 2018;32(11):1986‐1992. 29573483 10.1111/jdv.14948

